# PEG-Bottlebrush Stabilizer-Based Worm-like Nanocrystal Micelles with Long-Circulating and Controlled Release for Delivery of a BCR-ABL Inhibitor against Chronic Myeloid Leukemia (CML)

**DOI:** 10.3390/pharmaceutics14081662

**Published:** 2022-08-10

**Authors:** Huamin Liang, Fengming Zou, Liyi Fu, Qingwang Liu, Beilei Wang, Xiaofei Liang, Jing Liu, Qingsong Liu

**Affiliations:** 1 Anhui Province Key Laboratory of Medical Physics and Technology, Institute of Health and Medical Technology, Hefei Institutes of Physical Science, Chinese Academy of Sciences, Hefei 230031, China; 2 Hefei Cancer Hospital, Chinese Academy of Sciences, Hefei 230031, China; 3 Precision Medicine Research Laboratory of Anhui Province, Hefei 230088, China

**Keywords:** drug nanocrystal, stabilizer, micelle, BCR-ABL, chronic myeloid leukemia (CML)

## Abstract

Drug nanocrystals, one of most common drug delivery systems, enable the delivery of poorly water-soluble drugs with high drug loading and enhanced dissolution. The rapid clearance and uncontrolled drug release of drug nanocrystals limit their delivery efficiency and clinical application. Herein, an amphiphilic co-polymer, poly oligo(ethylene glycol) methacrylate-b-poly(styrene–co-4-formylphenyl methacrylate) (POEGMA-b-P (St-co-FPMA), PPP), characterized by a hydrophilic part with bottlebrush-like oligo(ethylene glycol) methacrylate (OEGMA) side chains, was synthesized as stabilizers to fabricate a high-drug-loading nanocrystal micelle (053-PPP NC micelle) using the chronic myeloid leukemia (CML) drug candidate N-(2-methyl-5-(3-(trifluoromethyl)benzamido)phenyl)-4-(methylamino)pyrimidine-5-carboxamide (CHMFL-ABL-053 or 053) as a model drug. The 053-PPP NC micelle was characterized and subjected to in vitro and in vivo studies. It featured a worm-like shape of small size, high drug loading (~50%), high colloidal stability, and controlled release in vitro. The presence of the 053-PPP NC micelle resulted in a long-circulation property and a much higher AUC. The 053-PPP NC micelle induced higher accumulation in the tumor tissues under multiple continuous administration. For in vivo efficacy, the 053-PPP NC micelle with a longer dosing interval (96 h), beneficial for improving patient adherence, demonstrated superiority to the 053-F127 NC. The proposed stabilizer PPP and the 053-PPP NC micelle with high drug loading enables drug delivery with long circulation and controlled release of drugs. It is also promising for the development of more efficient nanocrystal-based intravenous injection formulations for poorly water-soluble drugs. It might also offer new possibilities for potential clinical application of the CML candidate drug 053.

## 1. Introduction

Currently, cancer remains a global challenge. New molecular entity (NME) drugs, especially protein kinase inhibitors, have been one of major strategies against the challenge. To date, 68 small-molecule therapeutic protein kinase inhibitors have been approved by the Food and Drug Administration of the USA (US FDA) as of 10 December 2021 [1]. Numerous protein kinase inhibitor candidates are undergoing preclinical research or clinical trials. However, poor drug-like properties, such as low water solubility, short half-life, or fast metabolism, have been one of the major challenges, and limit the clinical translation of potential drug candidates. Approximately 40% of approved drugs, and nearly 90% of drug candidates, in the discovery pipeline are poorly water-soluble, and low water solubility has been one of the failures of most new drugs [2].

N-(2-methyl-5-(3-(trifluoromethyl)benzamido)phenyl)-4-(methylamino)pyrimidine-5-carboxamide (CHMFL-ABL-053 or 053), a drug candidate discovered by our group, is a highly selective BCR-ABL kinase inhibitor against chronic myeloid leukemia (CML) with high potency (GI_50_: ~14 nM against K562 cells) [3]. However, its poor water solubility and short half-life induces a low oral bioavailability (~24%), limiting its anticancer efficacy in vivo (TGI: 48.3% at 50 mg/kg, P.O.) [3]. Nanotechnology-based drug delivery enables intravenous administration of poorly water-soluble drugs without the use of toxic solubilizing agents such as organic solvents. In our previous work, we have made efforts to develop liposome- and polymeric prodrug micelle-based nanoparticles for the intravenous delivery of 053 with enhanced anticancer efficacy in vivo [4,5]. Nevertheless, the delivery systems with limited drug loading capacities (10–20%) presented in previous works need more excipients, causing potential safety burdens. Drug loading at a rate as high as possible is desired, which lessens the use of inactive excipients, and thus minimizes the possible safety issues associated with excipients.

Drug nanocrystals (NCs) are nanosized, solid drug particles with a protective layer of stabilizers on the surface. NCs are carrier-free colloidal dispersion systems with high drug loading capacities of up to 100% [6,7]. Nanosizing induces a high surface area, hence NCs exhibit increased saturated solubilities and dissolution rates. Thereby, NCs are promising delivery systems for poorly water-soluble drugs via oral or parenteral administration [8,9]. However, one of the major issues for their application in vivo is their rapid clearance from the blood circulation by the mononuclear phagocyte system (MPS) and reticuloendothelial system (RES), which results in insufficient drug accumulation in the target tissues [10]. In addition, the enhanced dissolution feature of NCs might cause uncontrolled dissolution/release into the bloodstream before reaching the target tissues, which hampers their ability to locally accumulate at the sites of disease [11]. As a result, many NCs exhibit pharmacokinetic profiles comparable to those of drug solutions [11,12,13,14]. These issues above make it a potential challenge to achieve the effective accumulation of NCs in target tissues, such as tumors. Surface coating and modification are expected to solve these issues. PEGylated coating has been used to control the drug release of NCs and enhance their stealth property to achieve long circulation [11]. In hematologic malignancies, tumor cells are free in the blood circulation, and in confined tumor sites such as the bone marrow and/or lymphoid tissues, in which the EPR effect can be exploited, are also present [15]. A long blood circulation duration for CML therapy is usually desired with nanoparticles, including NCs, to provide enough chance to interact with the free CML cells. Therefore, it is important to tune the NCs using optimal stabilizers to achieve a long circulation with controlled drug release. Poly(ethylene glycol) (PEG), a linear polymer known for its “stealth” properties, is commonly used for drug delivery to improve the pharmacokinetics of loaded drugs, enhance blood circulation, and reduce their recognition by the immune system [16,17]. Amphiphilic PEGylated polymers have been used for fabricating drug nanocrystal micelles to achieve targeted delivery to HER2-positive tumor cells with poly(ε-caprolactone)-co-poly(ethylene oxide) (PCL–PEG) conjugated with Herceptin [18], and to enhance in vivo circulation with methoxy polyethylene glycol-b-poly(L-lactide) (mPEG-PLA) [19]. In addition, functional PEGylated polymers have been developed for drug nanocrystals for special property, including PEG nanocages as non-sheddable stabilizers and redox-responsive stabilizers for drug nanocrystals [20,21]. However, its antigenicity, namely anti-PEG antibodies (APAs), is an increasing concern, which can cause enhanced clearance rates and loss of efficacy [16]. Poly(oligoethylene glycol) methacrylate (POEGMA), with a bottlebrush-like configuration, has been demonstrated to minimize the binding of APAs and retain the “stealth” property, and to improve the pharmacokinetics [16], and has been applied for drug delivery and nanomedicine [22,23].

**Figure 1:**
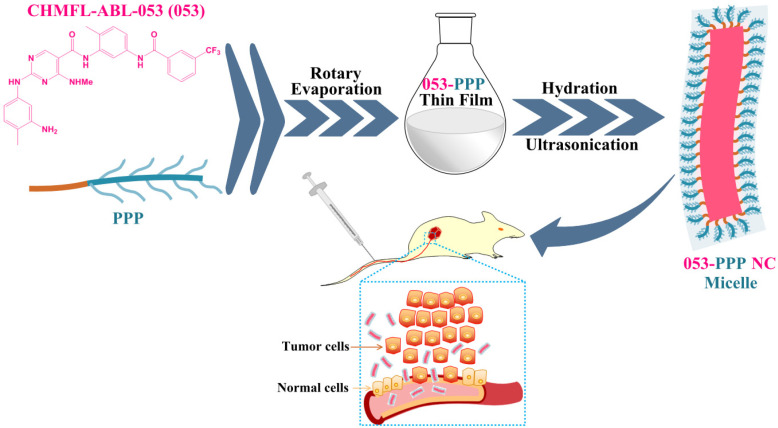
Schematic illustration for fabrication of the worm-like 053-PPP nanocrystal micelle, and its delivery to tumor tissues, in K562-tumor-bearing xenograft mice.

**Figure 2:**
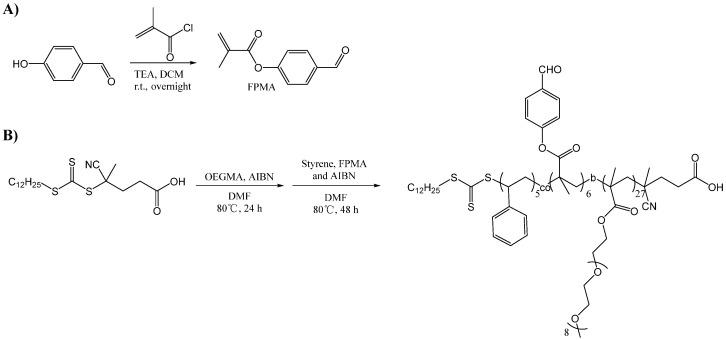
Schematic illustration of the synthesis of (**A**) the monomer 4-formylphenyl methacrylate (FPMA) and (**B**) the amphiphilic polymer POEGMA-b-P(St-co-FPMA) used as the stabilizers for the 053-nanocrystal micelles.

In this work, we designed and synthesized an amphiphilic polymer polyoligo (ethylene glycol) methacrylate-b-Poly (styrene–co-4-formylphenyl methacrylate) (POEGMA-b-P (St-co-FPMA), PPP), with bottlebrush-like OEGMA side chains, as stabilizers for fabricating drug nanocrystal micelles (Figure 1 and Figure 2). The introduction of styrene into the polymer can improve the hydrophobicity of the hydrophobic part of the polymer, and enhance the binding effect with drugs through the interaction between benzene rings, so as to improve the drug loading efficiency. The formylphenyl group was used to improve the affinity with drugs containing amino groups. The formed 053-PPP NC micelle was characterized in vitro and investigated in vivo, compared with the 053-F127 NC (fabricated using the commercial excipient Poloxamer Pluronic F127 as stabilizers). The polymerizing OEGMA brushes formed a micelle-like hydrophilic protective layer surrounding the surface of drug nanocrystals, and thus improved the pharmacokinetics (PKs) and release behavior of the NCs. Fabricated 053-PPP NCs had a high drug loading capacity (~50%) and displayed a more controlled release mode. Longer circulation and enhanced area under the curve (AUC) were achieved in rats. Improved anticancer efficacy in vivo was reached compared to free 053, even though only comparable efficacy to 053-F127 NCs was acquired.

## 2. Materials and Methods

### 2.1. Materials

The drug candidate N-(2-methyl-5-(3-(trifluoromethyl)benzamido)phenyl)-4-(methylamino)pyrimidine-5-carboxamide (CHMFL-ABL-053 or 053) was synthesized by our research group and the collaborator Hefei Cosource Pharmaceuticals Inc. (Hefei, China). The commercial amphiphilic block co-polymer Poloxamer Pluronic F127 (BASF) was ordered from a BASF distributor in China (Shenzhen Youpuhui Pharmaceutical Co., Ltd., Shenzhen, China). The Reversible Addition–Fragmentation Chain Transfer (RAFT) agent was synthesized according to the literature [24]. All other reagents and solvents were ordered from commercial sources, and were used as received unless specified otherwise. The Cell Counting Kit-8 (CCK8) (Best Bio, Shanghai, China) and K562 cells (ATCC, Manassas, VA, USA), and all other biological materials, were purchased from commercial sources.

### 2.2. Synthesis of POEGMA-b-P(St-co-FPMA)

The amphiphilic polymer POEGMA-b-P(St-co-FPMA) (PPP) used in this work was synthesized according to the protocol in our previous publication, with minor modifications [5]. Firstly, FPMA was synthesized. P-hydroxybenzaldehyde (2.00 g, 16.4 mmol) was dissolved in 40 mL dichloromethane (DCM), followed by addition of N, N-diisopropylethylamine (DIEA) (4.24 g, 32.8 mmol, 2 equivalent). Then, methacryloylchloride (1.89 g, 18.4 mmol, 1.1 equivalent) was added to the above mixture under a cooling condition by an ice-water bath (Figure 2A). The solution was stirred at room temperature overnight. After removing the solvent by reduced pressure evaporation, the residue was further purified by column chromatography with gradient elution from petroleum ether (PE): ethyl acetate (EA) = 60:1 to PE: EA = 50:1. 2.21 g transparent liquid was obtained with a yield of 70.9%. ^1^H NMR (500 MHz, CDCl_3_, δ) by the NMR BRUKER AVANCE NEO 500M: 10.01 (s, 1H), 7.95 (s, 2H), 7.31 (s, 2H), 6.40 (s, 1H), 5.83 (s, 1H), 2.09 (s, 3H) (Figure S1). Thereafter, POEGMA-b-P(St-co-FPMA) was synthesized. RAFT agent (100 mg, 0.247 mmol) was dissolved in 5 mL dimethylformamide (DMF) under argon atmosphere in a Schlenk bottle, followed by the addition of OEGMA (MW = 500) (4.95 g, 9.90 mmol, 40 equivalent) and azodiisobutyronitrile (AIBN) (12.1 mg, 0.074 mmol, 0.3 equivalent) (Figure 2B, Figure S1). The solution was stirred at 80 °C for 24 h, then styrene (206 mg, 1.98 mmol, 8 equivalent), FPMA (470 mg, 2.48 mmol, 10 equivalent), and AIBN (8.0 mg, 0.049 mmol, 0.2 equivalent) were added. After reacting at 80 °C for another 2 days, the solution was sealed in a dialysis bag and dialyzed against pure water for 24 h for purification to remove the organic solvents and the remaining monomers. Freeze-drying produced 3.5 g of yellow gel, with a yield of 91.0%. The molecular weight of the final co-polymer was calculated or characterized by NMR and gel permeation chromatography (GPC). Distribution of molecular weight was also characterized by GPC to provide the polydispersity index (PdI).

### 2.3. Preparation of 053 Nanocrystals and Nanocrystal Micelles

The 053-F127 nanocrystal (053-F127 NC) was prepared by a two-step method based on the previous work, with minor modifications [4]. In brief, primary 053-F127 NCs were fabricated with wet ball milling, followed by ultrasonication processing. The 053 powder was weighed and transferred into a 20 mL milling bowl, followed by the addition of 5 mL aqueous F127 solution (053/F127 weight ratio: 1:1). After adding 30 g of 0.5 mm zirconium oxide beads as milling pearls, grinding was performed for 10 milling cycles (each cycle consisted of 3 min milling followed by 15 min pause, allowing the bowl to cool down) at 1000 rpm using a planetary ball mill (Pulverisette 7 Premium, Fritsch Co., Idar Oberstein, Germany). Following this was ultrasonication processing for 3–5 min, with an amplitude of 30%, using the Branson Ultrasonics Sonifier™ 450 Cell Disruptor (BRANSON Ultrasonics Corporation, Danbury, CT, USA). After centrifuging for 15 min at 3000 rpm to remove the large particles, the acquired product, i.e., the final 053-F127 NC suspension, was stored at 4 °C until further use.

For 053-PPP nanocrystal micelles, a thin-film hydration method was applied (Figure 1). The drug candidate (053) powder and the stabilizer (PPP) at a ratio of 1:1 were dissolved in a mixed organic solvent of methanol and acetonitrile (8:5 *v*/*v*). The organic solvents were removed by rotary evaporation at 40 °C under vacuum to form the thin film of 053-PPP. After removing the residual organic solvents by maintaining for 1–2 h under high vacuum, the 053-PPP thin film was fully rehydrated with pure water, and then ultrasonicated for 3–5 min at an amplitude of 30% using the Branson Ultrasonics Sonifier™ 450 Cell Disruptor (BRANSON Ultrasonics Corporation, Danbury, CT, USA). The obtained suspension was centrifuged at 3000 rpm for 15 min to remove the large particles. The acquired supernatant consisting of the final 053-PPP nanocrystal micelles was stored at 4 °C until further use.

### 2.4. Characterization of 053 Nanocrystals and Nanocrystal Micelles

The size and morphology of the 053-F127 NCs and 053-PPP NC micelles were characterized by dynamic light scattering (DLS) analysis and transmission electron microscopy (TEM) imaging, respectively. The 053-F127 NCs and 053-PPP NC micelles were dispersed in pure water and determined by dynamic light scattering (DLS) using a Zetasizer Nano ZS90 system (Malvern Panalytical Ltd, Malvern, UK) to determine their particle diameter (Z-Average) and surface charge (zeta potential, ZP). In addition, the 053-F127 NCs and 053-PPP NC micelles were diluted in HEPES buffer at around pH 7.4 for zeta potential measurements to clarify the surface charge around physiological pH. The morphology of the 053-F127 NCs and 053-PPP NC micelles was imaged by TEM (HITACHI H-7650, Tokyo, Japan). In short, the 053-F127 NC and 053-PPP NC micelle samples were dropped onto a 400 mesh copper grid with formvar/carbon-supporting film. After drying in air, they were imaged by TEM at an accelerated voltage of 100 kV.

### 2.5. Entrapment Efficiency, Drug Loading, and Adsorbed Amount of Stabilizers on NCs

Entrapment efficiency (EE%) and drug loading (DL%) were determined via the methodology of the previous publication, with minor modifications [4]. The suspensions of the 053-F127 NCs and 053-PPP NC micelles and the supernatant following centrifugation for 15 min at 3000 rpm were analyzed by an Agilent 1260 HPLC system equipped with a UV detector at a wavelength of 274.5 nm using an Agilent TC-C18 column 250 mm × 4.6 mm, 5 μm. The HPLC analysis was performed at a flow rate of 0.8 mL/min with acetonitrile (0.1% *v/v* trifluoroacetic acid) and water (0.1% *v/v* trifluoroacetic acid) as the mobile phase of gradient elution at 20 °C. The general entrapment efficiency (*EE*%) of the 053-F127 NCs and 053-PPP NC micelles was calculated by Equation (1):

(1) $$EE\%=\frac{053\;conc.\;after\;centrifugation\;}{053\;conc.\;before\;centrifugation}\times 100$$

The general drug loading capacity (*DL*%) was calculated by Equation (2):

(2) $$DL\%=\frac{053}{053+stabilizer}\times EE\%$$

In order to calculate the adsorbed amount of stabilizers on the NC surface, some 053-F127 NCs and 053-PPP NC micelles suspensions were pipetted and centrifuged at a speed higher than 18,000 g for at least 2 h to ensure the NCs or NC micelles were sedimented. The sediment was collected and freeze-dried to quantify the ratio of drug and stabilizer by HPLC analysis using the same methods. The original ratio of stabilizers in the original 053 NC or NC micelle suspension was calculated according to the general drug loading. The ratio of adsorbed stabilizers was calculated based on the ratio of stabilizers in the sediment and the original ratio of stabilizers in the 053 NC or NC micelle suspension.

### 2.6. Colloidal Stability Study of 053 Nanocrystal and Nanocrystal Micelle Suspensions

The colloidal stability of the 053-F127 NC and 053-PPP NC micelle suspensions was investigated in vitro. The change in particle size (Z-Average) and ZP over time was monitored by DLS analysis.

### 2.7. In Vitro Release of 053 Nanocrystals and Nanocrystal Micelles

In vitro release was evaluated by assessing the dissolution of 053 NCs via light scattering technique using a DLS instrument Zetasizer Nano ZS90 (Malvern Instruments Ltd., Malvern, Worcestershire, UK) based on previous protocols, with minor modifications [4,25,26]. Based on the DLS analysis, the light scattering intensity displayed a linear correlation with the particle number and/or size. The derived count rate (i.e., absolute light scattering) could be monitored as a measure of particle dissolution (i.e., drug release). Pure water and 0.2% (*v*/*v*) tween 80 in H_2_O were used as release media. The derived count rates were monitored in real time using the DLS instrument immediately once the 053-F127 NCs or 053-PPP NC micelles were diluted in the prewarming release media (final 053 concentration: 10 μg/mL) in the DLS cuvette. The preset parameters for real-time DLS monitoring were as follows: (1) two runs at 2 s; (2) fixed position, 4.65 mm; (3) attenuator, 10; (4) temperature, 37 °C. The derived count rates of the samples minus those of the blank medium were plotted as a function of time. Based on the initial derived count rates at time zero, the derived count rates were further normalized into cumulative release rates, which was also plotted as a function of time.

### 2.8. In Vitro Cell Toxicity Assay

The K562 cell line used in this work was ordered from the American Type Culture Collection (ATCC, Manassas, VA, USA). The K562 cells were cultured in RPMI 1640 with 10% fetal bovine serum (FBS) (VivaCell, Shanghai, China) and 1% penicillin/streptomycin in a humidified incubator at 37 °C with 5% CO_2_. Firstly, K562 cells were seeded into 96-well plates with 100 μL of 1640 medium containing 3000 cells per well, and were incubated at 37 °C under 5% CO_2_. Subsequently, the cells in each well were treated with a series of concentrations of free 053 in DMSO, 053-F127 NCs, and 053-PPP NC micelles, respectively. Cell viability was determined using the Cell Counting Kit-8 (CCK8) (Best Bio, Shanghai, China) based on the manufacturer’s instructions. Subsequently, 10 μL of CCK8 solution was added to each well, and the absorbance of each well at a wavelength of 450 nm was measured using a microplate reader (iMARK, Bio-Rad, Hercules, CA, USA) after further incubation for 1 h at 37 °C. The absorbance was normalized and plotted as a function of drug concentration. The GI_50_ values were calculated using GraphPad Prism 8.0 (San Diego, CA, USA).

### 2.9. In Vivo Pharmacokinetics (PKs)

The in vivo profiling of free 053, 053-F127 NCs, and 053-PPP NC micelles were evaluated and compared using PK tests in male Sprague Dawley rats with individual weights of ~200 g (from the Laboratory Animal Center of Anhui Medical University, Hefei, China), approved by the Animal Ethics Committee of Hefei Institutes of Physical Science, Chinese Academy of Sciences, Hefei, China (approval code: DW-2019-20). The rats were housed in an air-conditioned room at 23 ± 2 °C and humidity of ~50%. After acclimatizing the facilities for 1 week, the rats were randomly divided into three groups (three per group): free 053, 053-F127 NC, and 053-PPP NC micelle. The rats were fasted for 12 h prior to drug administration. Free 053 (dissolved in 10% *v/v* DMSO in glucose 5%), 053-F127 NC, and 053-PPP NC micelle suspensions were administrated by tail vein injection at a dose of 1 mg/kg. Blood samples were collected in heparinized tubes at 2, 5, 15, 30 min and 1, 2, 4, 6, 9, 12, and 24 h (for all three groups). For the 053-PPP NC micelle group, blood was sampled at another three time points of 72, 96, and 120 h. The collected fresh blood samples were immediately processed in accordance with the following protocol: (1) collection of 100 μL of plasma by centrifuging the blood samples for 3 min at 6000 rpm and 4 °C, followed by storage at –80 °C for further analysis; (2) 20 μL of 200 ng/mL of caffeine (as an internal standard solution) was added to 100 μL of each plasma sample; (3) 400 μL of methanol was applied, vortexed for 5 min, and centrifuged at 14,000 rpm for 10 min; (4) the collected supernatants were quantified via LC-MS/MS analysis by loading 5 μL of supernatant. The collected data were analyzed by the non-compartment model using WinNonlin 8.1 software (Pharsight Corporation, Mountain View, CA, USA) to elucidate the PK parameters.

### 2.10. Tissue Distributions of 053

For the in vivo distributions, K562 xenograft nude mouse models were established to investigate the distribution of free 053, 053-F127 NCs, and 053-PPP NC micelles. In brief, approximately 6-week-old female Bal B/c nude mice (Beijing Vital River Laboratory Animal Technology Co. Ltd., Beijing, China) were housed in a specific pathogen-free animal facility for the animal tests, approved by the Animal Care Regulations of the Hefei Institutes of Physical Science Chinese Academy of Sciences (approval code: DW-2019-20). K562 cell suspension (1 × 10^7^ cells) mixed with Matrigel (BD Biosciences) at a ratio of 1:1 was inoculated into the right flank of nu/nu mice via subcutaneous injection. When the tumors grew up to 150–200 mm^3^, the mice were then randomly divided into nine groups (three mice per group) for: (1) 48 h after single dose of 40 mg/kg of free 053 (per os, PO), 053-F127 NC (intravenous, IV), or 053-PPP NC micelle (IV); (2) 96 h after a single dose of 40 mg/kg of free 053 (PO), 053-F127 NC (IV), or 053-PPP NC micelle (IV); (3) 48 h after three continuous doses of 40 mg/kg (once every other day, Q2D) of 053 (PO), 053-F127 NC (IV), or 053-PPP NC micelle (IV). The mice were sacrificed, and the main tissues and organs (including tumor, liver, heart, kidneys, lungs, spleen, and brain) were harvested at 48 h and 96 h after withdrawing blood samples. The collected tissues were then rinsed with PBS and weighed. Each tissue sample was mixed with saline at a ratio of 1:3 *w*/*v*, homogenized, and stored at −80 °C for further analysis. The further processing protocol prior to analysis was the same as for the plasma samples of PK described above: 5 μL of the prepared samples was loaded for LC-MS/MS analysis to quantify the 053 content. The withdrawn blood samples were processed and analyzed in accordance with the protocol for the rat PKs described above.

### 2.11. In vivo Anticancer Efficacy in K562 Xenograft Mouse Model

Female Bal B/c nude mice were used to establish K562 xenograft mouse models according to the protocol described above. When the tumors reached 150–200 mm^3^, the mice were randomly divided into seven groups (n = 5) for the evaluation of antitumor efficacy in vivo at 40 mg/kg: Vehicle, free 053 QD (Once a day, PO), 053-F127 NC QD (IV), 053-F127 NC Q2D (Once every other day, IV), 053-PPP NC QD (IV), 053-PPP NC Q2D (IV), and 053-PPP NC Q4D (once every 4 days, IV). During the treatment, body weight and tumor growth were measured daily. Tumor volumes (*V*) were calculated as follows (Equation (3)):

*V* (mm^3^) = [(*W*^2^ × *L*)/2], (3)

where width (*W*) and length (*L*) are defined as the lower and higher of two measurements, respectively. When the tumors of the vehicle group reached the limit of the animal ethical regulations, the test was stopped. All the mice were euthanized, and major tissues/organs were harvested for immunohistochemical (IHC) analysis, including HE, Ki-67, and TUNEL staining. Tumor growth inhibition (*TGI*) was calculated as follows (Equation (4)):

(4) $$TGI=\frac{W_{v}-W_{T}}{W_{v}}\times 100\%$$

where *W_v_* and *W_T_* are the tumor weight of the vehicle group and the treated groups.

### 2.12. Immunohistochemical (IHC) Analysis of Tumor Tissues

The harvested tumor tissues following the treatment were further evaluated by the IHC assay using three different staining methods, including hematoxylin–eosin (HE), Ki-67, and TdT-mediated dUTP nick end labeling (TUNEL) based on the previous protocol [4]. After fixing in 4% neutral buffered formalin and processing in paraffin, the tumor tissues were sectioned into 4 μm sections, and stained with HE, Ki-67 antibody, or TUNEL. The stained sections were imaged using an optical microscope at a magnification of 100×. The steps in detail for each staining method are not repeated here, but found in the previous publication [4].

### 2.13. In Vivo Safety Analysis

The safety of the treatment to the major organs was evaluated by the IHC assay with HE staining. The harvested major organs from the mice of each group were processed, stained by HE, and imaged following the protocol described above.

### 2.14. Statistical Analysis

The data are presented as the mean ± standard deviation (SD), unless otherwise specified. Statistical differences between two groups and multiple groups were analyzed using Student’s *t*-test. The difference was perceived as statistically non-significant (ns), significant (*) at *p* < 0.05, highly significant (**) at *p* < 0.01, and extremely significant (***) at *p* < 0.001 and (****) *p* < 0.0001.

## 3. Results and Discussion

### 3.1. Preparation and Characterization of 053-PPP NC Micelle

**Figure 3:**
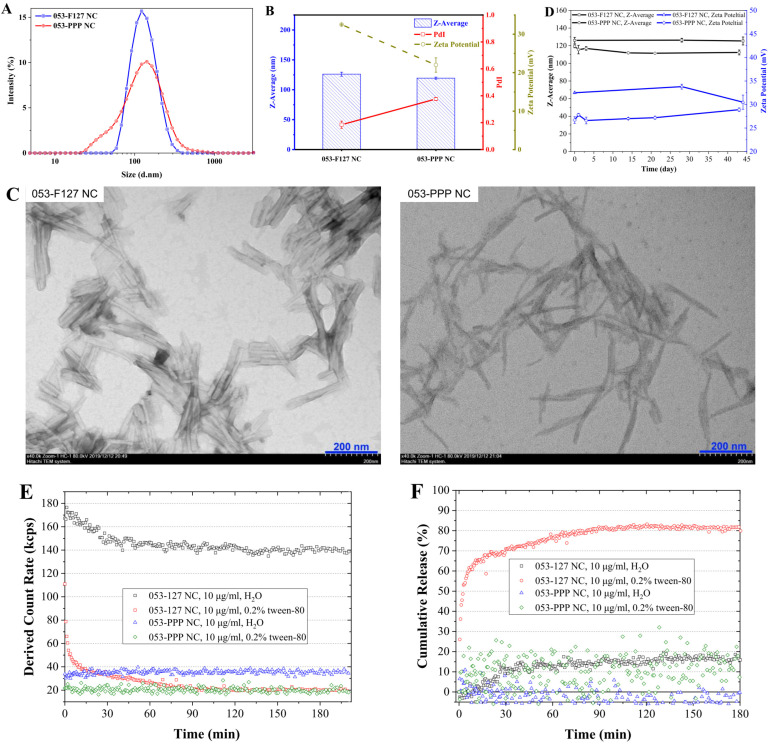
(**A**) Size distribution and (**B**) Z-average, PdI, and zeta potential of 053-F127 nanocrystal and 053-PPP nanocrystal micelles determined by dynamic light scattering (DLS). (**C**) Morphology images of 053-F127 nanocrystal and 053-PPP nanocrystal micelles from TEM at an accelerated voltage of 100 kV. (**D**) Stability study of 053-F127 nanocrystals and 053-PPP nanocrystal micelles by monitoring Z-average and Zeta Potential in water using dynamic light scattering (DLS). (**E**) Real-time monitoring of in vitro release behavior of 053-F127 NC and 053-PPP NC micelle, and (**F**) normalized cumulative release vs. time.

In this work, a worm-like 053-PPP nanocrystal micelle was fabricated and evaluated in vitro and in vivo (Figure 1). To achieve long circulation, an amphiphilic polymer POEGMA-b-P(St-co-FPMA) (PPP) was synthesized as stabilizers to fabricate the nanocrystal micelle of 053 (053-PPP NC micelle) (Figure 1). The PEG-like monomer (OEGMA) was first polymerized to a reversible addition–fragmentation chain transfer (RAFT) agent to form the hydrophilic part featured by multiple bottlebrush-like PEG side chains (Figure 2B). Thereafter, FPMA (Figure 2A) and styrene were added to the mixture for further co-polymerization to form the hydrophobic part. Based on the calculated integral values of the characteristic peaks for styrene (f), FPMA(j), OEGMA (c), and RAFT agent (a) in the ^1^H-NMR spectra, the final co-polymer was composed of ~27 units of OEGMA, ~6 units of FPMA, and 5 units of styrene (Figure S2). Thus, the average molecular weight (MW) of the polymer PPP was calculated to be 15566. The polymer PPP was further characterized by GPC. The measured MW was 34,701, with a good PdI of 1.103 (Figure S3). A significant difference between the MW values from NMR and GPC was observed. The possible reason for this is related to the structural difference between the standard polystyrene sample used for the standard curve for GPC, and our polymer. In this work, different protocols were used for fabricating the 053-PPP NC micelle and 053-F127 NC to achieve similar sizes. The 053-PPP NC micelles were fabricated by thin-film hydration combined with ultrasonication using PPP as stabilizers (Figure 1). In addition, Poloxamer F127 (Figure S4), a commercially available and common FDA-approved excipient composed of hydrophobic polypropylene oxide (PPO) and hydrophilic polyethylene oxide (PEO) chains, used as stabilizers for fabricating 053 drug nanocrystal (053-F127 NC) as a control by wet ball milling combined with ultrasonication for comparative study. Wet ball milling, probe sonication, and thin-film hydration are common methods used for drug nanocrystal or micelle preparation [6,19,27]. These methods are also usually combined to form drug nanocrystals of small size, and with a narrow size distribution [28]. The same method (thin-film hydration plus ultrasonication processing) as used for 053-PPP NC micelle preparation was attempted to prepare the 053-F127 NCs. Unfortunately, it was difficult to form nanocrystals less than 200 nm. Thus, a different protocol from that used for the 053-PPP NC micelle was applied for 053-F127 NC preparation. The 053-PPP NC micelle and 053-F127 NC were further characterized by DLS and TEM, and drug loading capacity was quantified by HPLC. A similar size was observed between the 053-PPP NC micelle and 053-F127 NC. Based on the appropriate fabricating protocol, the 053-PPP NC micelle had a small size (119.4 ± 1.7 nm) that was similar to that of 053-F127 NC (126.1 ± 3.4 nm) (Figure 3A,B). The size of the 053-PPP NC micelle was almost consistent with the previous publication, in which a size of 140 nm was observed for a paclitaxel-loaded PCL–PEG worm-like nanocrystal micelle [18]. The sizes of 053-PPP NC micelles and 053-F127 NC calculated from TEM images in Figure 3C were 89.5 and 74.8 nm, respectively (Table S2). The size values from TEM were smaller than, but comparable to, those from DLS. This is reasonable because the hydrodynamic diameter produced by DLS was usually higher than that by TEM due to an electric dipole layer adhering to its surface. The 053-F127 NC exhibited a rod-like shape, while the 053-PPP NC micelle displayed a worm-like shape with a higher aspect ratio (Figure 3C). However, a higher polydispersity index (PdI) value was observed for the 053-PPP NC compared with the 053-F127 NC. The reason for this might be associated with the worm-like shape of the 053-PPP NC. The higher aspect ratio might induce the inconsistency in specific shapes (e.g., curving) that was verified by the morphology from TEM imaging (Figure 3C). Therefore, the higher PdI was reasonable for the 053-PPP NC micelles. In pure water (pH 5–6), a highly positive zeta potential was observed for 053-F127 NC (32.5 mV) and 053-PPP NC micelle (22.0 mV) (Figure 3B). The 053-PPP NC micelle exhibited a lower zeta potential value in pure water, which was ascribed to the highly hydrophilic surface from multiple PEG side chains of the PPP co-polymer (Figure 3B). However, both of them showed a near-neutral surface charge with a zeta potential of 4.79 mV for 053-F127 NC and 4.23 mV for 053-PPP NC micelle in HEPES buffer around physiological pH 7.4 (Figure S5). The zeta potentials around physiological pH were not highly positive, which might contribute to long circulation with the highly hydrophilic property of PPP from the bottlebrush-like PEG chains. The worm-like morphology of micelles has potential to improve the rates of nanoparticles entering the tumor tissues [18]. The stability of the 053-PPP NC micelle and 053-F127 NC was further investigated by DLS analysis. Both showed an excellent stability in vitro. The diameter and zeta potential maintained were relatively consistent, with only a minor fluctuation after 40 days (Figure 3D).

The 053-PPP NC micelle and 053-F127 NC attained a high drug loading capacity of 48.09 ± 0.05% and 48.24 ± 0.21%, respectively. In our previous work, a pH-responsive 053-PPP prodrug was synthesized and assembled into the 053-PPP prodrug micelle with a drug loading of ~10% only [5]. The amount of adsorbed stabilizers on the NC surface was further characterized by high-speed centrifugation and HPLC analysis. Only a fraction of the stabilizers, 32.95 ± 0.10% for PPP and 47.00 ± 1.53% for F127, were adsorbed onto the NC surface (Table S1). This indicates that the stabilizers used during fabrication of 053 NC or NC micelles were sufficient to maintain the adsorbed stabilizer amount on the NC surface. In this work, the amphiphilic polymer PPP was utilized to load 053 and form worm-like micelles with high drug loading capacity. Under high drug loading, much more 053 molecules were deposited and crystalized into the core. On the surface, the amphiphilic PPP polymer formed a protective micelle layer. In order to know how controlled the drug releases from the NCs or NC micelles were, the drug release profiles of the 053-PPP NC micelle and 053-F127 NC were investigated in vitro. First, we tried to use dialysis plus HPLC analysis to investigate the release of the 053 NC or NC micelle. However, there was no characteristic peak of 053 detected by HPLC for the release medium following dialysis, which might be due to the poor water solubility and strong adsorption of 053 to the dialysis bag membrane. Therefore, the drug release was indirectly investigated via particle dissolution by DLS monitoring [26]. Based on this method, the light scattering property of a cloud of particles depends on the size and number of particles [25]. Following particle dissolution and drug release, the absolute light scattering intensity (derived count rate) should decrease correspondingly [4]. The derived count rate was normalized to cumulative release and plotted onto the release curves as a function of time (Figure 3E,F). The drug release investigation was conducted in both pure water and 0.2% tween 80. In pure water, the derived count rate of the 053-PPP NC micelle maintained almost stable, which indicates nearly no release (Figure 3E,F). The 053-F127 NC exhibited a slow release rate and a low release degree in water due to the poor water solubility of 053 (Figure 3E,F). In 0.2% tween 80, the 053-PPP NC micelle and 053-F127 NC showed rather different release profiles. The 053-F127 NC exhibited a fast release rate and reached more than 70% release within 30 min (Figure 3E,F). In contrast, the release curve of the 053-PPP NC micelle maintained almost constant values, and there was nearly no drug release observed within 3 h (Figure 3E,F). Based on these results, the 053-PPP NC micelle could release the loaded drugs in a controlled mode, which is beneficial for avoiding premature leakage before reaching the target sites. The bottlebrush-like POEGMA’s 3D hyperbranched structure can present highly dense oligoethylene glycol (EG) moieties [16,29,30]. The controlled drug release might be related to the highly dense oligoethylene glycol (EG) moieties of PPP, which sustain the drug release from the 053-PPP NC micelle.

### 3.2. In Vitro Antiproliferation

**Figure 4:**
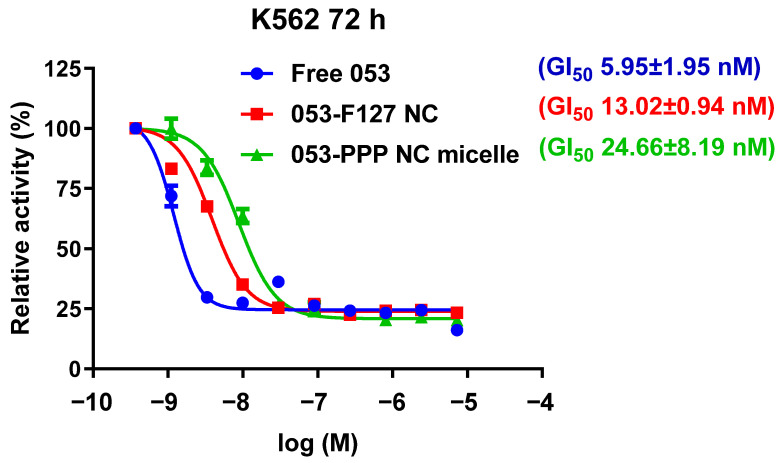
Cell viability/cytotoxicity of free 053, 053-F127 NC, and 053-PPP NC micelle in K562.

The in vitro antiproliferation activity of the 053-PPP NC micelle was assessed in K562 cells and compared with free 053 and the 053-F127 NC. Following exposure to the K562 cells, a dose-dependent antiproliferation effect and low GI_50_ values were acquired for all three 053 formulations (Figure 4). The 053-F127 NC and 053-PPP NC micelle exhibited a lower antiproliferation activity than free 053 (Figure 4), which might be ascribed to higher permeability of the dissolved free 053 molecules across the cell membranes. The activity of the 053-PPP NC, lower than that of the 053-F127 NC, might be related to its highly hydrophilic PEG surface, the less positive zeta potential, and slow drug release. Positive surface charge is usually beneficial to the internalization of nanoparticles into cells due to negative charge of the extracellular matrix. The less positive surface charge of the 053-PPP NC micelles may limit internalization, resulting in lower activity. Despite this, the 053-PPP NC micelle still had a relatively low GI_50_ value (24.66 nM). The difference in the GI_50_ of three 053 formulations was not exponential but comparable with each other.

### 3.3. PK and Tissue Distribution

**Figure 5:**
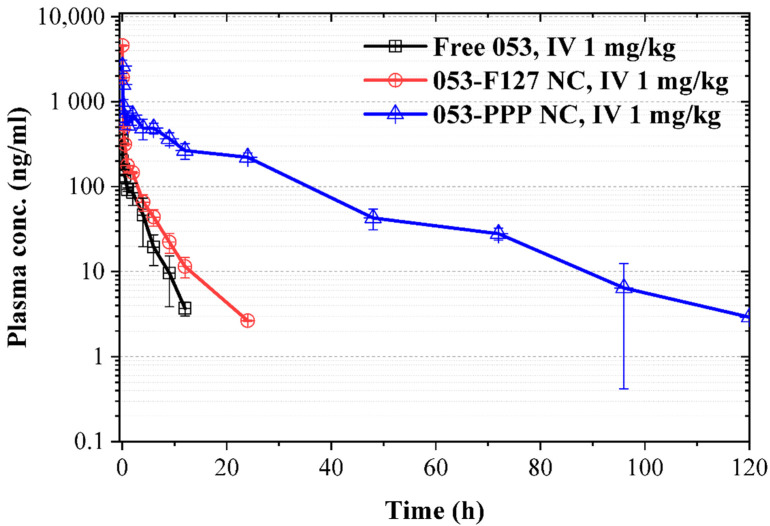
Pharmacokinetics (PK) in rats (n = 3). Free 053, 053-F127 NCs, and 053-PPP NC micelles were administered at a dose of 1 mg/kg via tail vein followed by withdrawal of blood samples at 2, 5, 15, 30 min and 1, 2, 4, 6, 9, 12, 24, 72, 96, 120 h.

The PK study was first performed on rats to evaluate the related parameters, half-life and area under the curve (AUC), via IV injection. The 053-PPP NC micelle exhibited much higher AUC and a longer half-life than free 053 and the 053-F127 NC (Figure 5 and Table 1). In contrast, the 053-F127 NC only displayed a slightly higher AUC and longer half-life than free 053 (Figure 5 and Table 1). The half-life increased from 2.02 h (free 053) and 4.44 h (053-F127 NC) to 14.19 h for the 053-PPP NC micelle (Table 1). At the same time, the 053-PPP NC micelle exhibited an almost 27-fold higher AUC than free 053, and a 9-fold higher AUC than the 053-F127 NC (Table 1). The PK results are ascribed to PEGylation, which is the most common strategy used to improve the biocompatibility of nanoparticles, and to achieve the stealth property and long circulation by avoiding rapid clearance of nanoparticles from the circulation system by the mononuclear phagocyte system (MPS) and reticuloendothelial system (RES) [17]. In this work, the improved PKs of the 053-PPP NC micelle may be ascribed to the hydrophilic POEGMA brushes of the bottlebrush-like PPP, which could minimize the binding of APAs and retain “nonfouling” properties that produce a “stealth” effect, such as for PEG. The controlled release from the PPP micelle layer may also contribute to the improved AUC and half-life. The enhanced exposure, which resulted from the greatly enhanced AUC and half-life, was expected to improve the bioavailability and further the antitumor efficacy in vivo. The highest C_max_ (4590 ng/mL), observed for the 053-F127 NC, demonstrated a significant initial burst release, remaining a challenge for translation [31]. In contrast, the 053-PPP NC micelle exhibited an appropriate C_max_ and slow decreasing plasma concentration, which is beneficial to assuring the therapeutic effect and safety.

**Figure 6:**
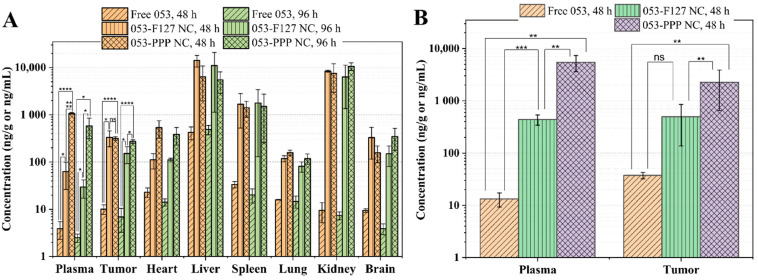
In vivo tissue distribution study using K562 xenograft mouse models (n = 3): (**A**) single dose at 48 h and 96 h post administration and (**B**) multiple dose (three doses, one every 48 h) 48 h post administration. (ns: statistically non-significant; *: statistically significant at *p* < 0.05; **: highly significant at *p* < 0.01; ***: extremely significant at *p* < 0.001; ****: extremely significant at *p* < 0.0001).

Tissue distribution of 053 in major organs and the tumor tissues was further evaluated following administration of different formulations in K562 xenograft tumor-bearing mice. The distribution of free 053 in the major organs/tissues at 48 h and 96 h post administration was similar except in the liver, which is consistent with the general distribution of free drugs throughout the body (Figure 6A). The long circulation property of the 053-PPP NC micelle was confirmed by it exhibiting the highest plasma concentrations at 48 h and 96 h, consistent with the PK results in rats (Figure 5 and Figure 6A and Table 1). The main accumulations of the 053-F127 NC and the 053-PPP NC micelle were observed in the RES organs, including the liver, spleen, and kidney (Figure 6A), associated with the common distribution behavior of nanoparticles. The 053-PPP NC micelle exhibited a slightly lower accumulation in the liver and spleen, which demonstrates the stealth effect produced by the highly PEGylated surface. In tumor tissues, the 053-F127 NCs and the 053-PPP NC micelles acquired comparable accumulation, but much higher than that of free 053, which is consistent with the EPR effect of nanoparticle in tumor tissues (Figure 6A). The concentration of 053 in the tumor tissues remained nearly unchangeable in the 053-PPP NC micelle group at 48 h and 96 h, while the free 053 and the 053-F127 NC exhibited a decrease between 48 h to 96 h (Figure 6A). The distribution in tumor and plasma was further investigated using multiple continuous administrations (three doses) to evaluate the cumulative distribution effect of drugs. The 053-PPP NC micelle still exhibited the highest plasma concentration under the multiple administrations, which, once again, confirms the long circulation behavior resulting from the stealth effect of the PEGylation surface. It is interesting that the distribution of the 053-F127 NC and the 053-PPP NC micelle in tumor tissue exhibited different behaviors between the multiple and single administrations. The 053-PPP NC micelle had a comparable accumulation in the tumor tissues to the 053-F127 NC following a single injection (Figure 6A). However, under multiple continuous administrations, the 053-PPP NC micelle accumulated in the tumor tissues at a significantly higher concentration than the 053-F127 NC (Figure 6B). This might be attributed to the higher cumulative effect from multiple administrations due to the better PK profile of the 053-PPP NC micelle, such as its longer circulation and lower RES clearance. The higher accumulation in the tumor tissues was expected to be beneficial for enhanced antitumor efficacy in vivo.

### 3.4. In Vivo Antitumor Efficacy and Safety Evaluation

**Figure 7:**
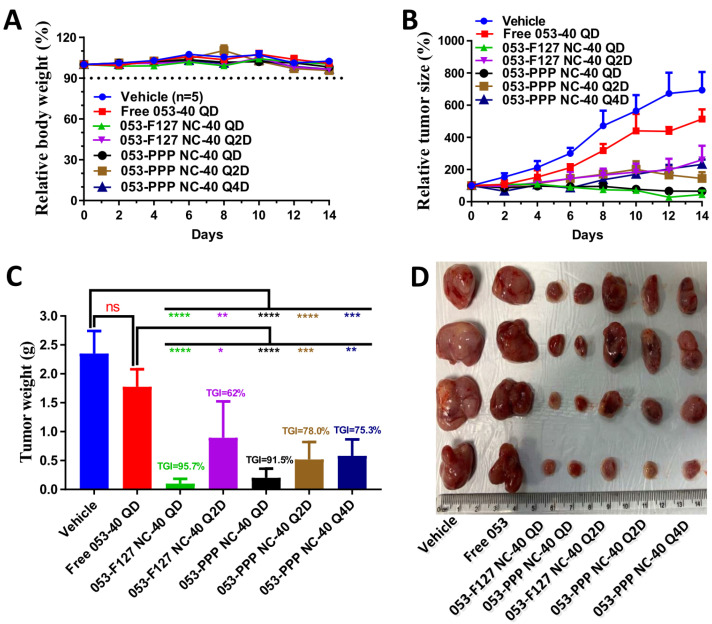
In vivo antitumor efficacy of free 053 (PO), 053-F127 NC (IV) and 053-PPP NC micelles (IV) using K562 xenograft mouse model. Seven groups (n = 5): (1) vehicle (PBS as control, IV injection daily), (2) free 053 40 mg/kg QD (free 053 powder dispersed in 0.5% methylcellulose/0.4% tween 80 in ddH2O, once a day), (3) 053-F127 NC 40 mg/kg QD (once a day, IV), (4) 053-F127 NC 40 mg/kg Q2D (once every other day, IV), (5) 053-PPP NC micelle 40 mg/kg QD (once a day, IV), (6) 053-PPP NC micelle 40 mg/kg Q2D (once every other day, IV), (7) 053-PPP NC micelle 40 mg/kg Q4D (once every 4 days, IV). Relative changes in body weight for each mouse group following the treatment were monitored (initial body weight was set as 100%) (**A**). Relative change in tumor size of xenograft mice during the treatment (initial tumor size was set as 100%) (**B**). Comparison of the final tumor weight for each group at the end of the treatment (TGI, tumor growth inhibition) (**C**). Representative pictures of tumors for each group at the end of the treatment (**D**). (ns: statistically non-significant; *: statistically significant at *p* < 0.05; **: highly significant at *p* < 0.01; ***: extremely significant at *p* < 0.001; ****: extremely significant at *p* < 0.0001).

In vivo antitumor efficacy of the 053-PPP NC micelle was evaluated in K562 xenograft mouse models and comparted with free 053 and the 053-F127 NC with different dosing regimens (40 mg/kg) at different dosing intervals. The free 053 did not show a significant difference in the tumor inhibition efficacy compared with the vehicle group. In contrast, all the groups treated with the 053-PPP NC micelle or the 053-F127 NC exhibited excellent tumor growth inhibition (TGI), with significant differences compared with the vehicle and the 053 group (Figure 7). The best tumor inhibition was acquired for dosing of 40 kg/mg QD of the 053-PPP NC micelle and 053-F127 NC, and the 053-PPP NC displayed a comparable TGI to the 053-F127 NC, with an identical significant difference (*p* < 0.0001). For dosing of 40 mg/kg Q2D, the 053-PPP NC micelle displayed certain superiority to the 053-F127 NC, even though there was statistically no significant difference observed between the two groups. The 053-PPP NC micelle acquired a higher mean TGI than the 053-F127 NC. Furthermore, the 053-F127 NC group showed a much higher standard deviation. It is interesting that the 053-PPP NC micelle Q4D achieved a comparable TGI to the Q2D (Figure 7). Therefore, at the same dose of 40 mg/kg, the 053-PPP NC micelle showed significant superiority to the 053-F127 NC due to its longer dosing interval (one dose every 96 h), which is beneficial for improving the patient adherence. These in vivo antitumor results are reasonable, and are the combined effect of the cellular activity, the PK, and the tissue distribution of the 053 formulations (Figure 4, Figure 5 and Figure 6). The higher tumor accumulation and the lower cellular activity for the 053-PPP NC micelle together generate the comparable TGI to the 053-F127 NC with Q2D and Q4D.

**Figure 8:**
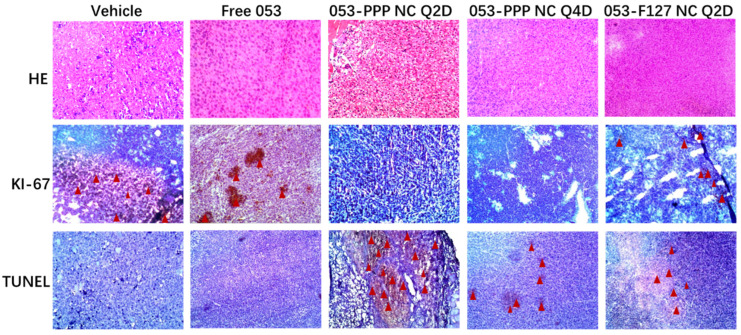
Representative micrographs of HE, Ki-67, and TUNEL staining assays of tumor tissues after treatment with free 053 formulations (PO), 053-F127 NCs and 053-PPP NC micelles (IV) at 40 mg kg^−1^ daily compared to the vehicle group.

After the treatments, the tumors were harvested and stained with HE, Ki-67, or TUNEL for IHC analysis. The Ki-67 staining assay was used for assessing tumor proliferation in vivo (Ki-67 positive expression, tan color; Ki-67 negative expression, blue color). The lowest Ki-67 expression level was observed in the two 053-PPP NC micelle-treated groups. In contrast, obvious positive Ki-67 expression was verified from staining images for the other three groups (see the arrows in Figure 8 Ki-67). The TUNEL staining assay was employed to evaluate the apoptosis of tumor cells in vivo, and the dark-brown color represents apoptotic cells. The most TUNEL positive staining was found in the group of 053-PPP NC micelle Q2D, and thus indicates the highest degree of apoptosis (see the arrows in Figure 8 TUNEL). These staining results suggest that the 053-PPP NC micelle group acquired the least cell proliferation and the highest apoptosis in the tumor tissues. This is almost consistent with the antitumor efficacy in vivo.

**Figure 9:**
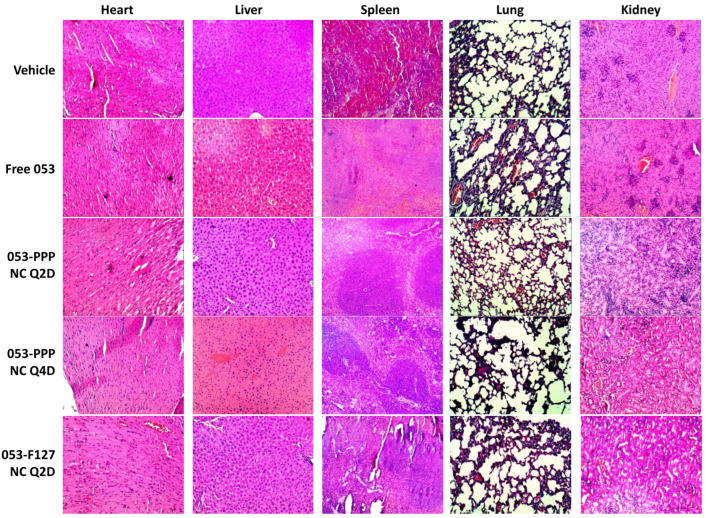
Evaluation of in vivo safety: representative micrographs of HE staining of histological sections of major organs (including the heart, spleen, kidneys, liver, and lungs). Magnification: 100×.

During the antitumor efficacy test, the weight of each mouse was monitored as one of measures to toxicity. There was no significant weight loss observed for any of the groups, and no significant difference was observed between the treated groups and the vehicle group. This indicates that the treatments with free 053 and the NC formulations were not detrimental to maintaining normal weight (Figure 7A). After the treatments, the major organs were harvested and stained by HE to evaluate the safety of the 053 formulations. None of the treated groups showed a significant difference compared to the vehicle group (Figure 9), validating the potential safety of the 053 formulations, including the 053-PPP NC micelle.

## 4. Conclusions

Based on the issues of drug nanocrystals such as rapid clearance and uncontrolled drug release, we designed and synthesized an amphiphilic co-polymer POEGMA-b-P(St-co-FPMA) (PPP), which had a hydrophilic part characterized by bottlebrush-like OEGMA side chains. A nanocrystal micelle (053-PPP NC micelle) was fabricated using the PPP as stabilizers and the CML drug candidate 053 as the model drug. The 053-PPP NC micelle exhibited a worm-like shape with small size, a high colloidal stability, and controlled release in vitro. Meanwhile, it had a higher drug loading activity (~50%) compared with the previous 053-PPP prodrug micelle (~10%). The 053-PPP NC micelle showed a long circulation property, a much higher AUC, and longer half-life due to the stealth effect of the PEGylated PPP micelle surface. The 053-PPP NC micelle exhibited a comparable accumulation in the tumor tissues to the 053-F127 NC following single dosing. In contrast, under multiple continuous administrations, the 053-PPP NC micelle exhibited a higher accumulation in the tumor tissues than the 053-F127 NC. For in vivo antitumor efficacy, the 053-PPP NC micelle did not indicate statistically significant difference in TGI at the dosing regimens of 40 mg/kg QD and 40 mg/kg Q2D, compared with the 053-F127 NC. However, the 053-PPP NC micelle dosed with 40 mg/kg Q4D showed a similar antitumor efficacy with the group Q2D, leading to significant superiority over the 053-F127 NC due to its longer dosing interval (one dose per 96 h), beneficial for improving the patient adherence. Base on the weight monitoring of mice and the HE staining assay, the 053-PPP NC micelle exhibited acceptable preliminary safety. In conclusion, as a proof of concept, this work proposes a stealth co-polymer with multiple PEG side chains and its corresponding 053 nanocrystal micelle with high drug loading, which enables drug delivery with long circulation and controlled drug release. It is also promising for developing intravenous injection formulation of poorly water-soluble drugs.

## Figures and Tables

**Table 1:** The PK parameters of free 053, 053-F127 NCs, and 053-PPP NC Micelles in rats (n = 3).

Parameters	Units	IV 1 mg kg^−1^		
		Free 053	053-F127 NC	053-PPP NC micelle
T_1/2_	h	2.02 ± 0.42	4.44 ± 0.65	14.19 ± 1.94
T_max_	h	0.03 ± 0.00	0.03 ± 0.00	0.03 ± 0.00
C_max_	ng/mL	364 ± 42	4590 ± 82	2617 ± 471
C_0_	ng/mL	555 ± 142	8116 ± 247	3658 ± 852
AUC_0_–_t_	h·ng/mL	499 ± 132	1518 ± 62	13346 ± 621
AUC_0_–_∞_	h·ng/mL	509 ± 132	1535 ± 60	13408 ± 645
V_z_	mL/kg	5870 ± 1329	4189 ± 713	1526 ± 180
Cl	mL/h/kg	2058 ± 552	652 ± 26	74.7 ± 3.6
MRT_0_–_t_	h	2.54 ± 0.55	2.55 ± 0.18	19.09 ± 2.27
MRT_0_–_∞_	h	2.78 ± 0.57	2.87 ± 0.16	19.57 ± 2.45

## Data Availability

Not applicable.

## Supplementary Materials

The following supporting information can be downloaded at: https://www.mdpi.com/article/10.3390/pharmaceutics14081662/s1. Figure S1: ^1^H-NMR spectrum of FPMA in CDCl_3_. Figure S2: ^1^H-NMR spectrum of polyoligo(ethylene glycol) methacrylate-b-Poly(styrene–co-4-formylphenyl methacrylate) (POEGMA-b-P (St-co-FPMA), PPP) in DMSO-d_6_. Figure S3: Gel Permeation Chromatography (GPC) spectra of POEGMA_27_-b-(PS_5_-r-PFPMA_6_). Figure S4: Molecular structure of Poloxamer F127, composed of hydrophobic polypropylene oxide (PPO) and hydrophilic polyethylene oxide (PEO). Figure S5: Zeta potential distribution of 053-F127 NC (upper) and 053-PPP NC micelle (lower) diluted in HEPES buffer, around physiological pH ~ 7.4. The zeta potential values, produced using Zetasizer Software 7.11, are 4.79 mV for 053-F127 NC and 4.23 mV for 053-PPP NC micelle, respectively. Table S1: Content of 053 and stabilizer in the freeze-dried sediments for the 053-PPP NC micelles and the 053-F127 NCs after centrifugation at a speed of higher than 18,000 *g* for at least 2 h, and the weight percent that the adsorbed stabilizers on the NC surface account for the feeding stabilizers for fabricating the 053 NCs. All the samples were analyzed by HPLC. Table S2: Size of 053-F127 NCs and 053-PPP NC micelles from the TEM images in Figure 3C analyzed by Image J (Final NC Size was calculated by the equation: Diameter = L/ln(L/W)).
