# Supplementary material for: PEG-Bottlebrush Stabilizer-Based Worm-like Nanocrystal Micelles with Long-Circulating and Controlled Release for Delivery of a BCR-ABL Inhibitor against Chronic Myeloid Leukemia (CML)
Source: Pharmaceutics. 2022 Aug 10;14(8):1662. doi: 10.3390/pharmaceutics14081662 (PMC9415161; doi:10.3390/pharmaceutics14081662)
Supplement: Supplementary file 1 [file pharmaceutics-14-01662-s001.zip › pharmaceutics-1766355-supplementary.pdf]

PEG-Bottlebrush Stabilizer-Based Worm-like Nanocrystal Micelles with Long-Circulating and Controlled Release for Delivery of a BCR-ABL Inhibitor against Chronic Myeloid Leukemia (CML)

Huamin Liang [1,2,†], Fengming Zou [1,2,†], Liyi Fu [1,2], Qingwang Liu [1,2], Beilei Wang [1,2], Xiaofei Liang [1,2], Jing Liu [1,2,3,*] and Qingsong Liu [1,2,3,*]

1 Anhui Province Key Laboratory of Medical Physics and Technology, Institute of Health and Medical Technology, Hefei Institutes of Physical Science, Chinese Academy of Sciences, Hefei 230031, China
2 Hefei Cancer Hospital, Chinese Academy of Sciences, Hefei 230031, China
3 Precision Medicine Research Laboratory of Anhui Province, Hefei 230088, China
* Correspondence: jingliu@hmfl.ac.cn (J.L.); qsliu97@hmfl.ac.cn (Q.L.)
† The authors contributed equally to this work.

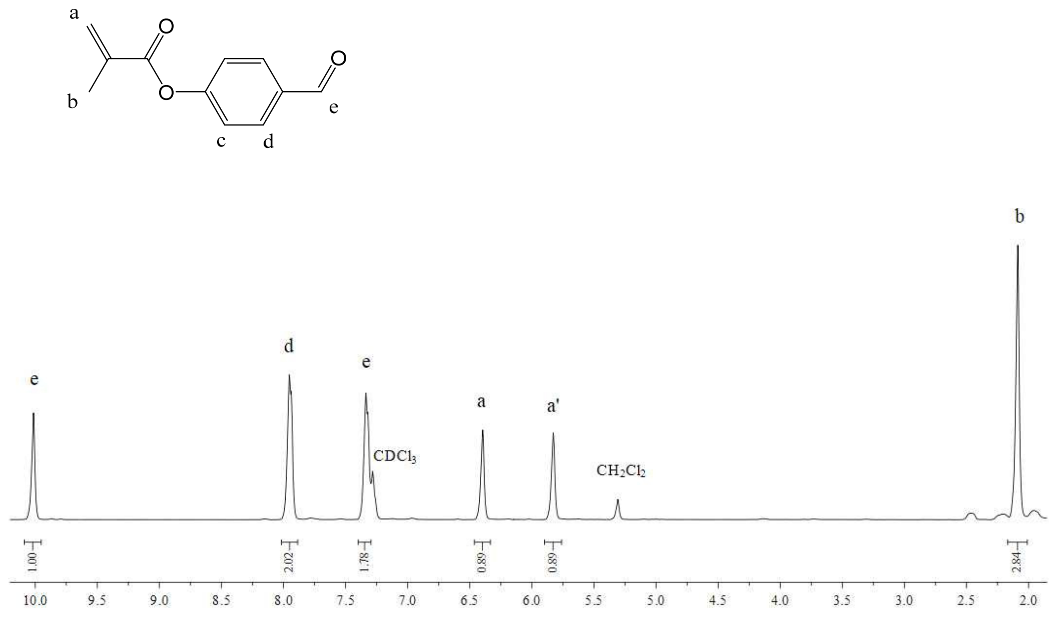

Figure S1 ^{1}H-NMR spectrum of FPMA in $CDCl_3$. The same letter (a–e) corresponds to the H atom in the structural formula and its counterpart characteristic peak of 1H-NMR. In addition, the positions of the characteristic peaks of the two H atoms at position a are different due to the different chemical environment, which are marked as a and a′.

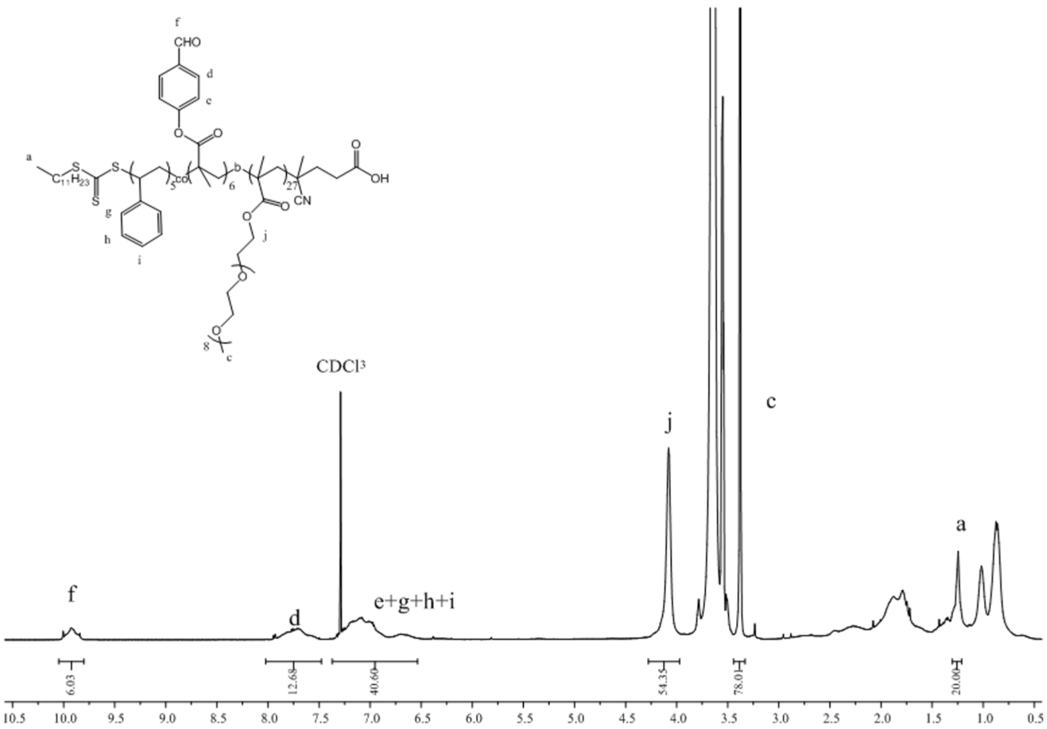

Figure S2 ^{1}H-NMR spectrum of polyoligo(ethylene glycol) methacrylate-b-Poly(styrene –co-4-formylphenyl methacrylate) (POEGMA-b-P (St-co-FPMA), PPP) in DMSO-d_6. Some characteristic H atoms on the main carbon chain and side chains and their corresponding characteristic peaks are represented and marked with the same letters (a–j).

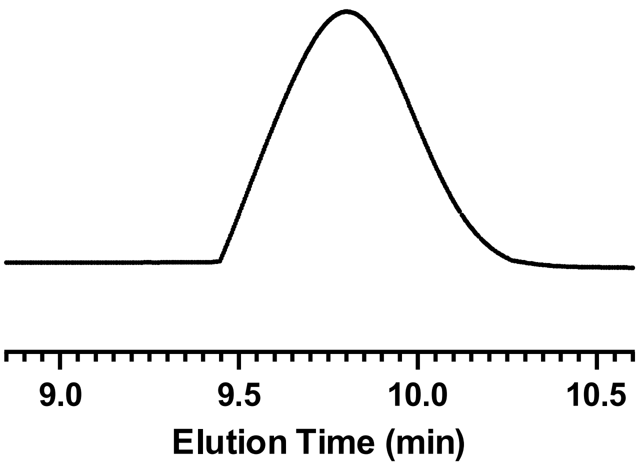

Figure S3 Gel Permeation Chromatography (GPC) spectra of $POEGMA_{27}$-b-(PS_5-r-$PFPMA_6$).

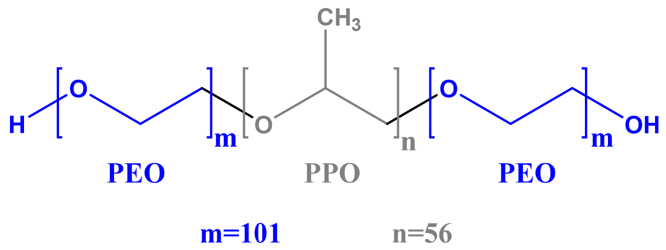

Figure S4 Molecular structure of Poloxamer F127, composed of hydrophobic polypropylene oxide (PPO) and hydrophilic polyethylene oxide (PEO).

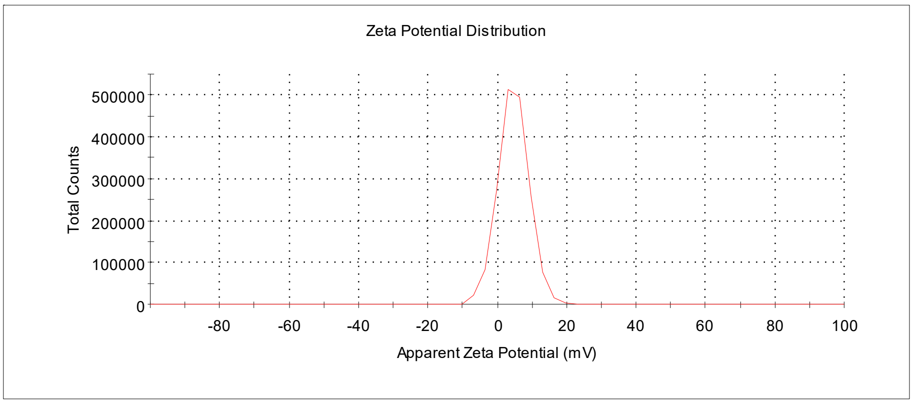

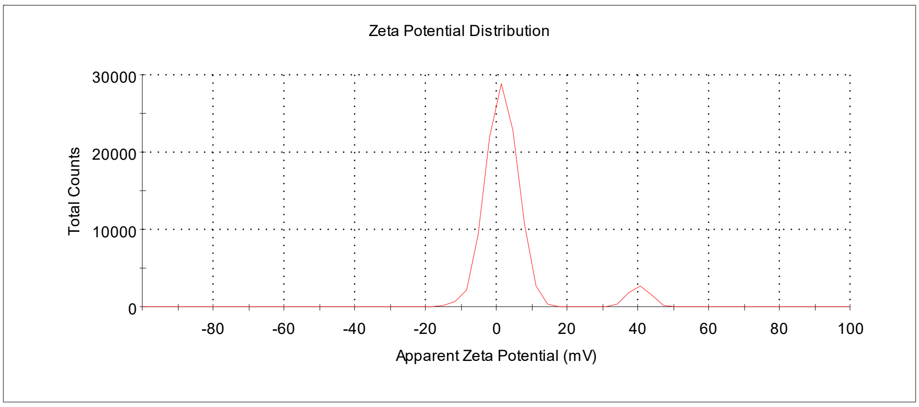

Figure S5 Zeta potential distribution of 053-F127 NC (upper) and 053-PPP NC micelle (lower) diluted in HEPES buffer, around physiological pH ~7.4. The zeta potential values, given by the ZetaSizer Software 7.11, are 4.79 mV for 053-F127 NC and 4.23 mV for 053-PPP NC micelle, respectively.

Table S1. Content of 053 and stabilizer in the freeze-dried sediments for the 053-PPP NC micelles and the 053-F127 NCs after centrifugation at a speed of higher than 18000 g for at least 2 h, and the weight percent that the adsorbed stabilizers on the NC surface account for the feeding stabilizers for fabricating the 053 NCs. All the samples were analyzed by HPLC.

Samples	Content of 053 in the Sediment	Content of Stabilizer in the Sediment	Percent of Adsorbed Stabilizers *
Freeze-dried sediment for 053-PPP NC micelles after centrifugation	82.90 ± 0.05%	17.1 ± 0.05% (PPP)	32.95 ± 0.10%
Freeze-dried sediment for 053-F127 NCs after centrifugation	75.67 ± 0.83%	24.33 ± 0.83% (F127)	47.00 ± 1.53%

** It is the weight percent that the adsorbed stabilizers on the NC surface account for the feeding stabilizers for fabricating the 053 NCs.*

Table S2. Size of 053-F127 NCs and 053-PPP NC micelles from the TEM images in Figure 3C analyzed by Image J (Final NC Size was calculated by the equation: Diameter = L/ln(L/W)).

Samples	Average Length (L) (nm)	Average Width (W) (Rod or Worm Diameter) (nm)	Calculated NC Size (nm)
053-PPP NC	227.6	17.9	89.5
053-127 NC	135.2	22.2	74.8
